# Influence of Work-to-Retirement Trajectories on Cognitive Outcomes in Later Life

**DOI:** 10.1093/geroni/igaf122.1299

**Published:** 2025-12-31

**Authors:** Yun Taek Oh

**Affiliations:** University of Nevada, Reno, Reno, Nevada, United States

## Abstract

Previous studies report that working longer is associated with better cognitive health. Yet, few studies evaluated the cognitive outcomes from various ways of working longer, such as remaining in career jobs longer, pursuing new careers, and gradually reducing work hours. While working in career jobs longer provides cognitive benefits, pursuing new careers in later life (i.e. encore careers) involves learning and training of different skills and knowledge that possibly can provide significantly more cognitive benefits by stimulating cognitive functionality. I use the Health and Retirement Study (HRS; n = 1,765) to evaluate how various work-to-retirement trajectories influence the cognitive outcomes of older workers during their later-phase of work lives (ages 60-71) and post-retirement lives (ages 70-81). I first use sequence analysis to categorize work-to-retirement trajectories into six groups. Then, I use latent growth modeling and competing risk analyses to examine the changes in cognitive health, separately by gender. The results show that working longer, regardless of making career transitions, is associated with better cognitive outcomes during the later-phase of work life (Slopes = 0.023 and 0.245 for pursuing new careers and taking phased exits respectively), and pursuing new careers in later life is associated with lower probability of getting dementia for men during post-retirement lives (Sub-distributional Hazard Ratio = 0.332). These results suggest that provision of learning and training opportunities for older workers, especially the skills and knowledge outside their career jobs, can contribute to preserving better cognitive health in later life.

